# Acute hospital-based services used by adults during the last year of life in New South Wales, Australia: a population-based retrospective cohort study

**DOI:** 10.1186/s12913-015-1202-8

**Published:** 2015-12-04

**Authors:** David E. Goldsbury, Dianne L. O’Connell, Afaf Girgis, Anne Wilkinson, Jane L. Phillips, Patricia M. Davidson, Jane M. Ingham

**Affiliations:** Cancer Research Division, Cancer Council New South Wales, Sydney, Australia; Sydney School of Public Health, University of Sydney, Sydney, Australia; School of Medicine and Public Health, University of Newcastle, Newcastle, Australia; Centre for Oncology Education and Research Translation (CONCERT), Ingham Institute for Applied Medical Research, South Western Sydney Clinical School, University of New South Wales, Sydney, Australia; School of Nursing and Midwifery, Faculty of Health, Engineering and Science, Edith Cowan University, Perth, Western Australia Australia; Faculty of Health, University of Technology Sydney, Sydney, Australia; School of Nursing, Johns Hopkins University, Baltimore, MD USA; Sacred Heart Health Service, St Vincent’s Health Network, Sydney, Australia; UNSW Australia, Faculty of Medicine, St Vincent’s Hospital Clinical School, Sydney, Australia

**Keywords:** End-of-life care, Terminal care, Palliative care, Hospitalisation, Emergency department presentations, Resource utilisation, Linked administrative health data

## Abstract

**Background:**

There is limited information about health care utilisation at the end of life for people in Australia. We describe acute hospital-based services utilisation during the last year of life for all adults (aged 18+ years) who died in a 12-month period in Australia’s most populous state, New South Wales (NSW).

**Methods:**

Linked administrative health data were analysed for all adults who died in NSW in 2007 (the most recent year for which cause of death information was available for linkage for this study). The data comprised linked death records (2007), hospital admissions and emergency department (ED) presentations (2006–2007) and cancer registrations (1994–2007). Measures of hospital-based service utilisation during the last year of life included: number and length of hospital episodes, ED presentations, admission to an intensive care unit (ICU), palliative-related admissions and place of death. Factors associated with these measures were examined using multivariable logistic regression.

**Results:**

Of the 45,749 adult decedents, 82 % were admitted to hospital during their last year of life: 24 % had >3 care episodes (median 2); 35 % stayed a total of >30 days in hospital (median 17); 42 % were admitted to 2 or more different hospitals. Twelve percent of decedents spent time in an ICU with median 3 days. In the metropolitan area, 80 % of decedents presented to an ED and 18 % had >3 presentations. Overall 55 % died in a hospital or inpatient hospice. Although we could not quantify the extent and type of palliative care, 24 % had mention of “palliative care” in their records. The very elderly and those dying from diseases of the circulatory system or living in the least disadvantaged areas generally had lower hospital service use.

**Conclusions:**

These population-wide health data collections give a highly informative description of NSWhospital-based end-of-life service utilisation. Use of hospital-based services during the last year of life was common, with substantial variation across sociodemographic groups, especially defined by age, cause of death and socioeconomic classification of the decedents’ place of residence. Further research is now needed to identify the contributors to these findings. Gaps in data collection were identified - particularly for palliative care and patient-reported outcomes. Addressing these gaps should facilitate improved monitoring and assessment of service use and care.

## Background

As with many developed countries around the world, the Australian population is expanding and ageing [1]. These demographic changes have resulted in increasing demands for health services at the end of life, but to date there is little comprehensive information about the hospital-based experiences of Australians at the end of life, particularly in the most populous state, New South Wales (NSW) [2]. Furthermore, no state-wide population-based studies have described emergency department (ED) presentations or time spent in intensive care at the end of life in NSW. This leaves a substantial information gap for those who need to plan and provide services for end-of-life care.

One previous population-based study in NSW described place of death for people who died from cancer [3], and a recent report described ED use after a cancer diagnosis and prior to death [4]. Another NSW study focused on hospital costs for decedents aged 65 years or more [5]. The only state-wide study in Australia that covered end-of-life hospital use (other than costs) for all decedents was about place of death, conducted in Western Australia for deaths between July 2000 and December 2002 [6]. Other studies focusing on hospital use at the end of life were restricted to specific diseases or age groups or surveys of carers of decedents [e.g. [7–11]]. Population-based studies in other countries have also focused on end-of-life care for specific diseases, particularly cancer [12]. Place of death has been reported for the whole population in many countries [e.g.[13–15]], however the substantial variation recorded by geography and causes of death underscores the importance of having comprehensive local information.

The aim of this study was to describe patterns of use of acute hospital-based health services by NSW adult residents during their last year of life using linked, routinely collected administrative health data. We present a number of measures based on previous studies [12] and discussed with key stakeholders in the health system, in response to an identified need for information relating to patients’ experiences at the end of life. These data will provide a baseline for future work related to end-of-life care. All adults who died in NSW in 2007 were included (as of May 2015 this was the most recent year for which cause of death information was available for all decedents).

## Methods

### Data sources and record linkage

This is a retrospective population-based record linkage study using a number of administrative health data collections, as described in detail previously [2]. Data were collated and analysed using privacy-preserving principles. From the NSW Register of Births, Deaths and Marriages (RBDM), we identified all people who died in NSW during the 2007 calendar year. Coded causes of death were obtained for these people from the Australian Bureau of Statistics (ABS) Mortality data. We also obtained information about their hospitalisations between January 2006 and December 2007 from the NSW Admitted Patient Data Collection (APDC), information on their presentations to EDs for the same period through the NSW Emergency Department Data Collection (EDDC), and information on any cancer diagnoses for these people between January 1994 and December 2007 from the NSW Central Cancer Registry (CCR). Probabilistic linkage of records in these data sets was carried out by the Centre for Health Record Linkage (CHeReL) using privacy-preserving methods [16]. The NSW Population and Health Services Research Ethics Committee approved the study and the linkage process.

As described previously, during the study period the EDDC contained information for 46 % of the 185 EDs in NSW, accounting for 81 % of ED attendances in the state [2]. We restricted our analysis of ED presentations to decedents from the “Greater Sydney Area” as we believe there is sufficiently complete information on their presentations to EDs. This area covered the Central Coast, Illawarra Shoalhaven, Nepean Blue Mountains, Northern Sydney, South Eastern Sydney, South Western Sydney, Sydney and Western Sydney Local Health Districts, accounting for 62 % of NSW adult deaths.

### Characteristics of decedents

Personal characteristics available from the data sources included age at death, sex, marital status, country of birth, need for interpreter service, and causes of death. Based on the local government area of residence at the time of death, decedents were assigned to a socioeconomic status quintile using the ABS index of relative disadvantage [17] and a category for accessibility to services as defined by the Accessibility/Remoteness Index for Australia (ARIA+) [18]. We restricted our analysis to people aged 18 years or more at death. Cause of death was taken from the ABS ‘underlying cause of death’ field but each person could also have up to 20 contributing causes of death recorded. Cause of death was available as a 4-character code using the International Classification of Diseases 10th revision (ICD-10).

### Measures of hospital-based service utilisation

We generated indicators of hospital-based service utilisation for each person during the 365 days prior to death, including the number and length of hospital admissions, the number of ED presentations, the amount of time spent in an intensive care unit (ICU), admissions for which the hospital record documented some aspect of palliative care, procedures recorded in hospital and place of death. To assess hospitalisations for cases, we aggregated hospital admission records with overlapping dates to represent one hospital episode. Each measure was selected from those reported previously [12] and in response to needs identified through consultation with health service leaders, administrators and policy makers. Data on the costs of care are not reported in our study due to the focus being on the patients’ experiences at the end of life.

Several fields in the APDC were used to create two indicators of use of palliative-related services during an admission. The first indicator captured people who were clearly documented as having been seen by a specialist palliative care team, based on admissions with a specific flag indicating that the person saw a palliative team, or having an admission to any of the five stand-alone inpatient hospices in NSW. The second indicator covered all identifiable admissions potentially related to palliative care and comprised all admissions captured in the first indicator, together with those where the service unit type was classified as a palliative care bed, admissions where the service category or service related group or a diagnosis code indicated palliative care (indicating palliative care was the reason for admission or a factor that could have impacted on the hospital stay), and admissions where the person was flagged as being referred to a palliative care team or palliative unit or an inpatient hospice. Thus the second indicator included individuals whose admission may have involved the delivery of palliative care. That stated, in this group it was not clear that the palliative care was delivered by a specialist palliative care service and may have been delivered by another medical team.

People were flagged as having been admitted to an ICU based on evidence in the APDC that a number of hours were spent in intensive care during an admission. The information provided did not allow us to determine whether the person was in an ICU at the time of death, or on the day of death.

Deaths in hospitals or EDs were identified using the arrival/separation status recorded in the APDC and EDDC. Deaths in hospices were only identifiable for the five stand-alone inpatient hospices in NSW that have individual institution codes. Hospice units or beds that are co-located within a hospital do not have a separate institution code so deaths occurring in them have the hospital recorded as the place of death. The CCR also recorded place of death as hospital, inpatient hospice, nursing home, or the person’s home, but such detailed data were not available for deaths that were not noted in the CCR. For those with a CCR record who were not flagged in the APDC or EDDC as having died in a hospital or inpatient hospice, place of death was taken as that recorded in the CCR.

### Data analysis

Measures of hospital utilisation from the APDC were analysed for all adult decedents and those related to ED presentations were restricted to decedents from the Greater Sydney Area. These measures were selected in consultation with key stakeholders in the health services, who expressed an interest in identifying frequent users of the health system based on reasonable and clinically relevant cut-points, rather than identifying factors potentially associated with a person having one additional unit of health service use (e.g. one more day in hospital). The indicators of hospital utilisation during the last year of life were: being in the highest quartile of the distribution for the number of hospital episodes (>3), being in the highest quartile for number of days in hospital (>42 days), being in the highest quartile for number of ED presentations (>3), the two indicators for palliative care, admission to ICU, being admitted to (or having an ED presentation at) three or more different hospitals, and dying in a hospital or inpatient hospice. Key patient characteristics associated with each of these indicators were examined using multivariable logistic regression including: age at death (18–59, 60–79, 80–89, 90+ years), sex, country of birth (Australia, other), accessibility/remoteness of place of residence (major cities, inner regional, outer regional/remote/very remote), socioeconomic status quintile of place of residence, and cause of death (diseases of the circulatory system, cancer, other causes, unknown). A sensitivity analysis was undertaken for each of these measures by excluding all people who died from external causes (including falls, self-harm, traffic accidents), as this group is likely to have a very different health care experience at the end of life. For analyses of ED presentations, accessibility/remoteness of place of residence was restricted to major city and inner regional areas.

Marital status and need for interpreter service were only available from APDC and EDDC records, so this information was not available for decedents who did not have linked records in either of these data sets. We therefore could not include these variables in the logistic regression models for hospital/ED use. Similarly, for decedents who did not have linked records in the ABS Mortality data or CCR we could obtain demographic information only if they had a record in the APDC or EDDC, and thus the 3 % of decedents with unknown cause of death were excluded from the logistic regression analyses. All analyses were carried out in SAS version 9.3 (SAS Institute Inc, Cary, North Carolina, US).

## Results

There were 45,761 people aged 18 years or more who died in NSW in 2007. We excluded 12 decedents who had hospital admission records or ED presentation records that could not be reconciled with their date of death (e.g. multiple admissions after date of death), as these cases were likely to reflect incorrect dates recorded or incorrect linkages. For the remaining 45,749 decedents, median age at death was 80 years (interquartile range [IQR] 70–87). The most common underlying causes of death were diseases of the circulatory system (34 % of all deaths) and neoplasms (29 %) (Table 1). As described previously [2], the next most common disease group (diseases of the respiratory system) accounted for only 8 % of deaths, and so in the analysis all other known causes of death have been grouped together.

**Table 1:** Characteristics of decedents aged 18 years or more in NSW in 2007 (*n* = 45,749)

	No. of deaths	% of deaths
Age at death		
18–59	5722	13
60–79	15,723	34
80–89	16,354	36
90+	7950	17
Sex		
Female	22,422	49
Male	23,117	51
Unknown	210	0.5
Country of birth		
Australia	33,862	74
Other	11,536	25
Unknown	351	1
Marital status		
Never married	4120	9
Married (including de facto)	18,262	40
Widowed	14,281	31
Separated/Divorced	2949	6
Unknown	6137	13
Interpreter required		
No	36,522	80
Yes	1651	4
Unknown	7576	17
Place of residence^a^		
Major cities	30,901	68
Inner regional	10,990	24
Outer regional	3175	7
Remote / Very remote	221	0.5
Unknown	462	1
Socioeconomic status^b^		
Most disadvantaged quintile	9045	20
Quintile 2	10,207	22
Quintile 3	10,169	22
Quintile 4	7878	17
Least disadvantaged quintile	7943	17
Unknown	507	1
Cause of death		
Diseases of the circulatory system	15,500	34
Cancer	13,441	29
Other causes	15,226	33
Cause of death not available	1582	3

^a^Based on Australian Bureau of Statistics’ Accessibility Remoteness Index for Australia

^b^Using population-based quintiles of the Australian Bureau of Statistics’ index of relative disadvantage

### Hospital episodes of care

**Fig. 1:**
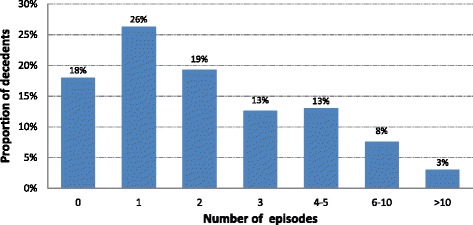
Hospital episodes of care in the last year of life for decedents aged 18 years or more in NSW in 2007 (*n* = 45,749)

**Fig. 2:**
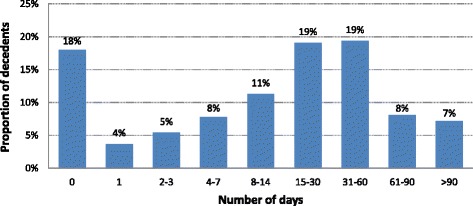
Total number of days in hospital in the last year of life for decedents aged 18 years or more in NSW in 2007 (*n* = 45,749)

Eighty-two percent of decedents (*n* = 37,497) had a hospitalisation recorded in the APDC during their last year of life. After aggregating hospital admission records with overlapping dates to represent one hospital episode, the median number of episodes for each person was 2 (IQR 1–3) (Fig. 1) and the median length of each episode was 3 days (IQR 1–10). During the last year of life, the median time in hospital was 17 days per person (IQR 3–42) and 35 % of decedents spent more than 30 days in hospital (Fig. 2). Among 37,567 decedents aged 65 years and older, 83 % had at least one hospitalisation in their last year of life.

**Fig. 3:**
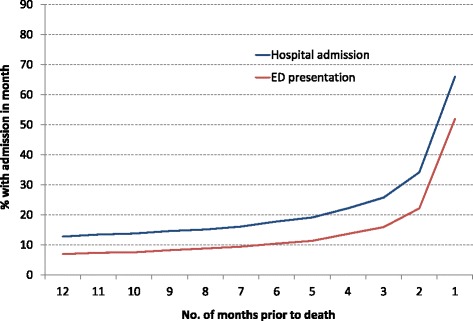
Proportion of decedents in hospital (*n* = 45,749) or presenting to ED (*n* = 28,484) in each month preceding death, for decedents aged 18 years or more in NSW in 2007

In terms of proximity to death, 59 % of decedents had at least one hospital admission in their last week of life, 66 % in the last month, 73 % in the last 3 months and 77 % in the last 6 months of life (Fig. 3). Restricting analyses to those resident in the Greater Sydney Area only (the region for which ED presentations were comprehensively recorded), these proportions each increased by 1 %.

ED: Emergency Department. The source of referral for hospital admissions in the last year of life was an ED for 41 % of hospital episodes, 39 % were referred by another medical practitioner, 7 % were from outpatient facilities, 3 % came from another hospital or a nursing home and 11 % were from other or unknown sources.

After examining the associations across the key patient characteristics, there were higher odds of having >3 hospital episodes for people who died from cancer compared with diseases of the circulatory system (adjusted odds ratio [aOR] 3.18, 95 % confidence interval [CI] 3.00–3.37), while it was less common for decedents aged 90 years or more or 80–89 years compared with 60–79 years (aOR 0.39, 95 % CI 0.36–0.42 and aOR 0.69, 95 % CI 0.65–0.72 respectively) (Table 2). Similarly, spending more than 42 days in hospital in the last year of life was more common among people who died from cancer compared with diseases of the circulatory system (aOR 2.17, 95 % CI 2.06–2.30), while it was less common for decedents aged 90 years or more or 18–59 years compared with 60–79 years (aOR 0.65, 95 % CI 0.61–0.70 and aOR 0.76, 95 % CI 0.71–0.82 respectively) (Table 2). Other statistically significant differences were observed but as the odds ratios were relatively close to 1 they were not considered to be clinically important. Excluding day-only admissions for dialysis (recorded for 1 % of decedents) did not change these results.

**Table 2:** Patient characteristics related to >3 hospital episodes, length of stay >42 days and time in ICU in the last year of life for people aged 18 years or more in NSW in 2007 (*n* = 45,749)^a^

		>3 hospital episodes (24 %)		>42 days in hospital (25 %)		Spent time in ICU (12 %)	
	No. of deaths	*n* (% within category)	Adjusted OR (95 % CI)	*n* (% within category)	Adjusted OR (95 % CI)	*n* (% within category)	Adjusted OR (95 % CI)
Age at death			*p* < 0.0001		*p* < 0.0001		*p* < 0.0001
18–59	5722	1660 (29)	0.92 (0.86–0.99)	1344 (23)	0.76 (0.71–0.82)	1018 (18)	1.06 (0.97–1.15)
60–79	15,723	4979 (32)	1.00 (reference)	4580 (29)	1.00 (reference)	2608 (17)	1.00 (reference)
80–89	16,354	3350 (20)	0.69 (0.65–0.72)	4051 (25)	0.92 (0.88–0.97)	1413 (9)	0.46 (0.42–0.49)
90+	7950	878 (11)	0.39 (0.36–0.42)	1389 (17)	0.65 (0.61–0.70)	235 (3)	0.14 (0.12–0.17)
Sex			*p* < 0.0001		*p* = 0.02		*p* < 0.0001
Female	22,422	4645 (21)	1.00 (reference)	5313 (24)	1.00 (reference)	2189 (10)	1.00 (reference)
Male	23,117	6222 (27)	1.19 (1.14–1.25)	6051 (26)	1.05 (1.01–1.10)	3085 (13)	1.14 (1.07–1.21)
Unknown	210	0 (0)	Not included	0 (0)	Not included	0 (0)	Not included
Country of birth			*p* = 0.01		*p* < 0.0001		*p* = 0.58
Australia	33,862	8084 (24)	1.00 (reference)	8566 (25)	1.00 (reference)	3840 (11)	1.00 (reference)
Other	11,536	2783 (24)	0.93 (0.89–0.99)	2798 (24)	0.89 (0.85–0.94)	1432 (12)	0.98 (0.91–1.05)
Unknown	351	0 (0)	Not included	0 (0)	Not included	2 (1)	Not included
Place of residence^b^			*p* = 0.04		*p* < 0.0001		*p* < 0.0001
Major cities	30,907	7527 (24)	1.00 (reference)	7843 (25)	1.00 (reference)	3797 (12)	1.00 (reference)
Inner regional	10,990	2492 (23)	0.92 (0.87–0.98)	2571 (23)	0.89 (0.84–0.94)	1090 (10)	0.75 (0.69–0.82)
Outer regional/Remote/Very remote	3396	830 (24)	0.97 (0.89–1.07)	921 (27)	1.06 (0.97–1.16)	373 (11)	0.69 (0.61–0.79)
Unknown	462	18 (4)	Not included	29 (6)	Not included	14 (3)	Not included
Socioeconomic status^c^			*p* < 0.0001		*p* = 0.01		*p* < 0.0001
Most disadvantaged quintile	9045	2234 (25)	0.90 (0.83–0.97)	2309 (26)	0.97 (0.89–1.04)	1338 (15)	1.23 (1.11–1.36)
Quintile 2	10,207	2439 (24)	0.83 (0.77–0.89)	2485 (24)	0.89 (0.83–0.96)	1091 (11)	0.86 (0.78–0.95)
Quintile 3	10,169	2251 (22)	0.78 (0.72–0.84)	2503 (25)	0.92 (0.86–0.99)	1065 (10)	0.82 (0.74–0.90)
Quintile 4	7878	1874 (24)	0.85 (0.79–0.92)	1938 (25)	0.91 (0.85–0.98)	802 (10)	0.75 (0.68–0.83)
Least disadvantaged quintile	7943	2043 (26)	1.00 (reference)	2088 (26)	1.00 (reference)	958 (12)	1.00 (reference)
Unknown	507	26 (5)	Not included	41 (8)	Not included	20 (4)	Not included
Cause of death			*p* < 0.0001		*p* < 0.0001		*p* < 0.0001
Circulatory system	15,500	2341 (15)	1.00 (reference)	2922 (19)	1.00 (reference)	1540 (10)	1.00 (reference)
Cancer	13,441	5465 (41)	3.18 (3.00–3.37)	4685 (35)	2.17 (2.06–2.30)	1514 (11)	0.79 (0.73–0.85)
Other known cause	15,226	2797 (18)	1.19 (1.12–1.26)	3447 (23)	1.26 (1.19–1.33)	2032 (13)	1.21 (1.12–1.30)
Cause not known	1582	264 (17)	Not included	310 (20)	Not included	188 (12)	Not included

*OR* odds ratio adjusted for all other factors in table, *CI* confidence interval

^a^These groups represent the top quartiles of the distributions for hospital episodes (>3) and length of stay in hospital (>42 days) during the last year of life

^b^Based on Australian Bureau of Statistics’ Accessibility Remoteness Index for Australia

^c^Using population-based quintiles of the Australian Bureau of Statistics’ index of relative disadvantage

### Presentations to Emergency Departments

Among the 28,484 adults resident in the Greater Sydney Area who died in 2007, 80 % (*n* = 22,915) had at least one ED presentation record during their last year of life. The median number of ED presentations was 1 (IQR 1–3) and the median duration of each ED presentation was 6 hours (IQR 4–9). Thirty percent of decedents presented to an ED in their final week of life and 52 % presented in their final month (Fig. 3). Three percent of decedents were dead on arrival at their first and only presentation to ED during their last year of life. For adults resident in the Greater Sydney Area, 88 % had at least one hospital admission or ED presentation recorded during the last 12 months of life and 65 % during the final week.

Having >3 ED presentations (18 % of decedents) was more common for those from the most compared with the least disadvantaged areas (aOR 1.80, 95 % CI 1.63–1.99) and for people who died from cancer or other causes compared with circulatory system disease (aOR 1.33, 95 % CI 1.23–1.45 and aOR 1.30, 95 % CI 1.20–1.40 respectively), while it was less common for decedents aged 90 years or more compared with 60–79 years (aOR 0.61, 95 % CI 0.55–0.68) (Table 3). Decedents from inner regional areas were more likely to have >3 ED presentations (aOR 1.18, 95 % CI 1.04–1.34) than those from major cities. There were no other important associations with the key patient characteristics (Table 3). The results were similar when ED presentations where the decedent was dead on arrival were excluded.

**Table 3:** Patient characteristics related to >3 ED presentations in the last year of life for people aged 18 years or more in the Greater Sydney Area of NSW in 2007 (*n* = 28,484)^a^

		>3 ED presentations (18 %)	
	No. of deaths	*n* (% within category)	Adjusted OR (95 % CI)
Age at death			*p* < 0.0001
18–59	3624	775 (21)	1.04 (0.94–1.14)
60–79	9607	1968 (20)	1.00 (reference)
80–89	10,269	1751 (17)	0.87 (0.81–0.94)
90+	4984	586 (12)	0.61 (0.55–0.68)
Sex			*p* < 0.0001
Female	14,208	2263 (16)	1.00 (reference)
Male	14,276	2817 (20)	1.18 (1.11–1.26)
Country of birth			*p* < 0.0001
Australia	18,924	3191 (17)	1.00 (reference)
Other	9473	1889 (20)	1.14 (1.07–1.22)
Unknown	87	0 (0)	Not included
Place of residence^b^			*p* = 0.01
Major cities	26,830	4725 (18)	1.00 (reference)
Inner regional	1651	355 (22)	1.18 (1.04–1.34)
Unknown	3	0 (0)	Not included
Socioeconomic status^c^			*p* < 0.0001
Most disadvantaged quintile	4394	1002 (23)	1.80 (1.63–1.99)
Quintile 2	4808	1044 (22)	1.69 (1.53–1.87)
Quintile 3	4392	767 (17)	1.35 (1.22–1.50)
Quintile 4	6958	1251 (18)	1.40 (1.27–1.53)
Least disadvantaged quintile	7884	1016 (13)	1.00 (reference)
Unknown	48	0 (0)	Not included
Cause of death			*p* < 0.0001
Circulatory system	9462	1378 (15)	1.00 (reference)
Cancer	8547	1743 (20)	1.33 (1.23–1.45)
Other known cause	9626	1805 (19)	1.30 (1.20–1.40)
Cause not known	849	154 (18)	Not included

^a^This group represents the top quartile of the distribution for ED presentations (>3) during the last year of life

^b^Based on Australian Bureau of Statistics’ Accessibility Remoteness Index for Australia

^c^Using population-based quintiles of the Australian Bureau of Statistics’ index of relative disadvantage

### Admissions to intensive care units

Twelve percent (*n* = 5274) of all adult decedents spent time in an ICU, with 87 % of these having only one ICU admission. Of those who had an ICU admission, the median time spent in ICU during the last year of life was 3 days (IQR 2–7). Six percent (*n* = 2890) of decedents died during a hospital admission that included time in ICU, although we cannot determine from the available data if death occurred while in the ICU. Of the key patient characteristics of interest, having time in an ICU was less common for decedents aged 90 years or more or 80–89 years compared with 60–79 years (aOR 0.14, 95 % CI 0.12–0.17 and aOR 0.46, 95 % CI 0.42–0.49 respectively) and those from outer regional or remote areas compared with major cities (aOR 0.69, 95 % CI 0.61–0.79) (Table 2). Compared with those who died from circulatory system diseases, people who died from other causes were more likely to be admitted to an ICU (aOR 1.21, 95 % CI 1.12–1.30) (Table 2).

### Palliative care

As described earlier, we developed two indicators for hospital-based or inpatient hospice-based palliative care utilisation in the last year of life: the first comprised inpatient admissions to inpatient hospices or hospital admissions flagged with “saw palliative team”; the second comprised any mention of current or future palliative care (including hospital and inpatient hospice admissions captured in the first indicator) or referral to an inpatient hospice (Table 4). The first indicator captured 15 % of decedents during their last year of life, with just over half of these having only one hospital admission with palliative care recorded and 24 % were captured by the second indicator. The available data did not allow a clear determination of the level of palliative care expertise provided to decedents or the intensity of their palliative care needs.

**Table 4:** Indicators of palliative care during hospital admissions in the last year of life for decedents aged 18 years or more in NSW in 2007 (*n* = 45,749)

	Number of deaths (%)	Cumulative deaths (%)^a^
Palliative care indicator 1		
Admitted to an inpatient hospice	2170 (5)	2170 (5)
“Saw palliative team”	6609 (14)	6909 (15)
Palliative care indicator 2		
Admitted to an inpatient hospice	2170 (5)	2170 (5)
“Saw palliative team”	6609 (14)	6909 (15)
Palliative care bed (unit type)	353 (1)	6954 (15)
Service category “Palliative”	5995 (13)	8228 (18)
Diagnosis “Palliative care”	9842 (22)	10,652 (23)
Referred to palliative team/palliative unit/hospice	1938 (4)	10,777 (24)

^a^Cumulative total of admissions/deaths by row going down the table

Having a palliative care hospital admission was most common for people who died from cancer. For indicator 1, the odds were more than 10 times higher for people who died from cancer compared to circulatory system diseases (aOR 12.88, 95 % CI 11.81–14.05), and this indicator was less commonly present for decedents from outer regional or remote areas compared with major cities (aOR 0.36, 95 % CI 0.31–0.42) and decedents aged 90 years or more compared with aged 60–79 years (aOR 0.54, 95 % CI 0.49–0.61). Having any palliative-related record in the last year of life (Indicator 2) was again more common for people who died from cancer compared with circulatory system diseases (aOR 14.89, 95 % CI 13.88–15.96), while it was less common for decedents aged 90 years or more compared with 60–79 years (aOR 0.56, 95 % CI 0.51–0.61) and decedents from the middle socioeconomic quintile compared with the least disadvantaged quintile (aOR 0.72, 95 % CI 0.66–0.79) (Table 5).

**Table 5:** Patient characteristics related to hospital-based palliative care in the last year of life for people aged 18 years or more in NSW in 2007 (*n* = 45,749)

		Palliative care indicator 1 (15 %)		Palliative care indicator 2 (24 %)	
	No. of deaths	*n* (% within category)	Adjusted OR (95 % CI)	*n* (% within category)	Adjusted OR (95 % CI)
Age at death			*p* < 0.0001		*p* < 0.0001
18–59	5722	1068 (19)	0.89 (0.82–0.97)	1601 (28)	0.86 (0.80–0.93)
60–79	15,723	3384 (22)	1.00 (reference)	5109 (32)	1.00 (reference)
80–89	16,354	1936 (12)	0.73 (0.68–0.78)	3160 (19)	0.74 (0.70–0.79)
90+	7950	521 (7)	0.54 (0.49–0.61)	907 (11)	0.56 (0.51–0.61)
Sex			*p* = 0.45		*p* = 0.53
Female	22,422	3121 (14)	1.00 (reference)	4877 (22)	1.00 (reference)
Male	23,117	3788 (16)	0.98 (0.92–1.04)	5900 (26)	0.98 (0.93–1.04)
Unknown	210	0 (0)	Not included	0 (0)	Not included
Country of birth			*p* < 0.0001		*p* < 0.0001
Australia	33,862	4649 (14)	1.00 (reference)	7650 (23)	1.00 (reference)
Other	11,536	2258 (20)	1.28 (1.20–1.37)	3125 (27)	1.17 (1.10–1.24)
Unknown	351	2 (1)	Not included	2 (1)	Not included
Place of residence^a^			*p* < 0.0001		*p* < 0.0001
Major cities	30,907	5538 (18)	1.00 (reference)	7809 (25)	1.00 (reference)
Inner regional	10,990	1060 (10)	0.46 (0.42–0.50)	2201 (20)	0.75 (0.70–0.81)
Outer regional/Remote/Very remote	3396	297 (9)	0.36 (0.31–0.42)	746 (22)	0.81 (0.72–0.90)
Unknown	462	14 (3)	Not included	21 (5)	Not included
Socioeconomic status^b^			*p* < 0.0001		*p* = 0.01
Most disadvantaged quintile	9045	1409 (16)	1.27 (1.15–1.40)	2231 (25)	1.02 (0.93–1.12)
Quintile 2	10,207	1317 (13)	0.86 (0.78–0.95)	2238 (22)	0.76 (0.69–0.82)
Quintile 3	10,169	1405 (14)	0.88 (0.80–0.97)	2133 (21)	0.72 (0.66–0.79)
Quintile 4	7878	1358 (17)	1.02 (0.93–1.12)	2039 (26)	0.99 (0.90–1.07)
Least disadvantaged quintile	7943	1403 (18)	1.00 (reference)	2109 (27)	1.00 (reference)
Unknown	507	17 (3)	Not included	27 (5)	Not included
Cause of death			*p* < 0.0001		*p* < 0.0001
Circulatory system	15,500	679 (4)	1.00 (reference)	1227 (8)	1.00 (reference)
Cancer	13,441	5253 (39)	12.88 (11.81–14.05)	7841 (58)	14.89 (13.88–15.96)
Other known cause	15,226	896 (6)	1.30 (1.17–1.44)	1564 (10)	1.29 (1.19–1.39)
Cause not known	1582	81 (5)	Not included	145 (9)	Not included

*Palliative Care Indicator 1* admitted to an inpatient hospice or had a hospital admission flagged with “saw palliative team”, *Palliative Care Indicator 2* any mention of current or future palliative care (including Indicator 1) or referred to an inpatient hospice, *OR* odds ratio adjusted for all other factors in table, *CI* confidence interval

^a^Based on Australian Bureau of Statistics’ Accessibility Remoteness Index for Australia

^b^Using population-based quintiles of the Australian Bureau of Statistics’ index of relative disadvantage

### Changes in location of hospital care

Forty percent of decedents were admitted to only one hospital during their last year of life (including 20 % who had only one hospital admission); 26 % were admitted to two different hospitals and 15 % were admitted to three or more different hospitals. Of the key patient characteristics of interest, being admitted to three or more different hospitals was more common for people who died from cancer (28 %) compared with people who died from diseases of the circulatory system (10 %) (aOR 2.94, 95 % CI 2.75–3.14) and those from the least disadvantaged areas (19 %) compared with the most disadvantaged areas (15 %) (aOR 1.36, 95 % CI 1.24–1.49), while it was less common for decedents aged 90 years or more (7 %) compared with 60–79 years (20 %) (aOR 0.47, 95 % CI 0.43–0.52) and people born outside of Australia (13 %) compared with Australian-born (16 %) (aOR 0.70, 95 % CI 0.65–0.74). The results were similar when admissions to inpatient hospices were excluded.

Combining hospital admissions and ED presentations for decedents from the Greater Sydney Area, 40 % were at only one hospital, 29 % at two different hospitals and 19 % at three or more different hospitals. The key patient characteristics associated with being at three or more hospitals in the Greater Sydney Area were similar to those for being admitted to three or more hospitals except for place of residence where it was more common for people from inner regional areas (23 %) compared to those living in a major city (19 %) (aOR 1.33, 95 % CI 1.17–1.51).

### Place of death

Death was recorded as occurring in hospital for 52 % of decedents, including 4 % who died while in the ED. Another 4 % were recorded as dying in one of the five recorded inpatient hospices, 5 % were dead on arrival to ED, while 0.4 % (*n* = 182) were discharged from hospital on the date of their death and thus were not recorded as dying in hospital. After including information from the CCR, place of death was still unknown for around one-third of all decedents but we can conclude that they most likely died outside of hospital, inpatient hospice or an ED. Restricting analyses to decedents from the Greater Sydney Area (where recording of presentations to ED was most complete), the distribution of place of death was similar to that for all adults in NSW. Fifteen percent of decedents aged 18–59 years (845/5722) were dead on arrival at the ED, compared with 5 % of decedents aged 60–79 years (796/15,723), 2 % aged 80–89 years (361/16,354) and only 1 % of decedents aged 90 years or more (73/7950).

Of the key patient characteristics of interest, dying in a hospital or inpatient hospice was more common for people who died from cancer (72 %) compared with people who died from diseases of the circulatory system (46 %) (aOR 2.64, 95 % CI 2.51–2.78), while it was less common for decedents aged 90 years or more (39 %) or 18–59 years (56 %) compared with 60–79 years (66 %) (aOR 0.46, 95 % CI 0.43–0.49 and aOR 0.68, 95 % CI 0.64–0.73 respectively).

### Deaths due to external causes

Five percent (*n* = 2140) of adult deaths were due to external causes (including falls, self-harm, traffic accidents). These causes were most common for decedents aged 18–59 years, with 20 % of this group dying from external causes compared with only 2–3 % in other age groups. In all other sociodemographic groups, 3–6 % of decedents died from external causes. Compared with those dying from all other causes, lower proportions of people who died from external causes used all forms of hospital care except for ICU where the proportions were the same (12 %).

When deaths from external causes were excluded from the analyses, estimates of the odds ratios increased for each measure of hospital-based services used by 18–59 year old decedents compared with those aged 60–79 years, and the estimated odds ratios for “other causes” versus circulatory system deaths also increased slightly. For example, after excluding people who died from external causes there was no longer a significant difference between decedents aged 18–59 years and those aged 60–79 years dying in hospital or inpatient hospice (aOR 1.00, 95 % CI 0.93–1.07). All other odds ratios for those aged 18–59 years compared with those aged 60–79 years were closer to unity except for time in ICU, where the odds ratio increased to 1.26 (20 % vs 17 %, 95 % CI 1.15–1.37). The exclusion of external causes of death made very little difference to the remaining associations examined.

### Procedures during hospital admissions

In addition to surgical and other clinical procedures, allied health interventions were recorded in the APDC. Overall there were 2988 different procedures recorded for people during their last year of life. The procedures recorded for the greatest number of people in the year prior to death were the allied health interventions of physiotherapy (54 % of decedents), social work (41 %) and occupational therapy (35 %), while the most common clinical “procedure” was a brain CT scan (23 %). Physiotherapy was the most common intervention, recorded in 51,537 (29 %) admissions, followed by dialysis in 35,238 (20 %) admissions, although dialysis was only recorded for 1 % of decedents, who on average had it recorded at 57 admissions each.

### Final hospital admissions

The median length of stay for the final hospitalisation prior to death (but not necessarily ending in death) was 9 days (IQR 3–20), with 35 % of episodes being longer than 14 days and 15 % being longer than 30 days. Among the 37,497 decedents with at least one hospital admission during their last year of life, their final admission ended on the date of death for 68 %, a further 13 % had an admission ending up to 30 days before death and another 8 % had a hospital admission that ended up to 3 months prior to death.

Among the people who died in hospital, the hospital episode that ended in death had a median length of 9 days and was longer than 30 days for 16 %. Having more than 30 days in hospital immediately prior to death was more common for decedents from outer regional, remote or very remote areas (22 %) compared with those from major cities (15 %) (aOR 1.74, 95 % CI 1.53–1.97) and those who died from cancer (19 %) or other causes (16 %) compared to those who died from diseases of the circulatory system (13 %) (aOR 1.66, 95 % CI 1.52-1.82 and aOR 1.31, 95 % CI 1.20-1.44 respectively). There were no other important associations with the key patient characteristics of interest.

Twelve percent of decedents died during a hospital episode flagged with palliative care indicator 1 (i.e. in an inpatient hospice or flagged with “saw palliative team”) and 18 % died during an episode that had mention of “palliative care”. The corresponding proportions were highest for people who died from cancer: 31 and 46 % respectively, compared to 3 and 6 % respectively for deaths from diseases of the circulatory system.

## Discussion

### Main findings

There was substantial variation in the hospital-based end-of-life experiences for different groups of decedents. For example: hospitalisations and records of hospital-based palliative care were more frequent for those dying from cancer compared to other common causes; ED presentations were more common for decedents from more disadvantaged areas; and hospitalisations, ED presentations and time in intensive care were less common for decedents aged 90 years or more.

Over one-third (35 %) of decedents spent more than 30 days in hospital in their last year of life. The proportions in hospital and numbers of hospitalisations in this study are similar to those reported in previous Australian studies for subgroups of decedents, with around 80 % admitted to hospital in their last year of life [5, 7], more hospital use for those who died from cancer [8] and very elderly decedents having fewer hospital admissions [7]. Population-based studies from other developed countries have reported similar levels of hospitalisation [19–24] or trends by age [20, 25] or cause of death [19, 24–26]. Over 40 % of decedents were admitted to two or more different hospitals, which, together with a previous USA study that reported a median of three healthcare transitions in the last 3 months of life [24], highlights the potential value of reliable information flow between hospitals for individual patients.

Given that people close to death might be more likely to encounter EDs at a time of profound symptom burden, data on ED presentations are essential to provide a comprehensive picture of the end-of-life experience. This is highlighted by the finding that 30 % of people who died in the Greater Sydney Area presented to the ED in the last week of life and 52 % presented in the final month. We found the numbers of ED presentations were higher for people from lower socioeconomic areas and those who died at a younger age. A recent review of ED use by cancer patients at the end of life reported similar results [27]. It has been suggested that people in areas of greater disadvantage might use the ED as a form of GP service, although this has been refuted by others [8, 28–31]. Overall the proportion of people presenting to an ED in the last year of life was similar to previous studies [8, 32]. Due to the incomplete coverage of the EDDC we could not examine the effect of living in rural or remote areas on ED presentations as analyses were restricted to those resident in the Greater Sydney Area.

It is important to note the constraints on our understanding of the use of palliative care services due to the current approach to data collection for this area of care in NSW. That stated, as has been reported elsewhere [e.g. [25, 33–38]], the evidence we have that relates to both the use and the mention of hospital-based palliative care services suggests these services were most commonly provided for people who died from cancer. This could be due to more established patterns of referral to specialised palliative care for cancer patients compared to people with other illnesses, along with there often being a better understood disease trajectory for cancer compared to others [35, 39]. Our data and that of others, however, raises questions about the reasons behind the disparities in relation to the use and mention of palliative care services for decedents who died from cancer versus those who died from a non-cancer illness, and whether access to and provision of palliative care for people dying from non-cancer causes is appropriately aligned with their needs [35, 36, 38, 40]. We also found that use and mention of hospital-based palliative care was less common among very elderly decedents and those not in major cities, as reported in a previous Western Australian study that also included community-based palliative care [33]. A recent UK-based review also described potentially inequitable provision of palliative care to various subgroups of decedents, such as those with non-cancer diseases and the very elderly [38]. Again, further work is needed to align these findings with a better understanding of the palliative care needs of this population.

Information on admissions to an inpatient hospice was limited, as it was only possible, using the existing data collection methods, to identify five stand-alone inpatient hospices that have their own unique institution code recorded in the APDC, all located in the Sydney metropolitan area. Inpatient hospice and palliative care beds and units are available at a number of other facilities in NSW, but there is no comprehensive method of distinguishing between admissions to these beds and admissions to other wards. Thus, while our results did indicate that inpatient hospice admissions were less common for people living in remote areas, it is not possible to accurately report on whether there are indeed fewer admissions to inpatient hospice or palliative care beds in these areas. This problem speaks to the importance of the need for refinement of current approaches to data collection.

Time in an ICU in the last year of life was most common for younger decedents, perhaps suggesting more ICU services were being directed towards people with the most years of life to gain. ICU admissions were less common for people who died from cancer, potentially due to these decedents often having a less precipitous decline in health towards the end of life and potentially having a stage of illness that resulted in their condition being less amenable to ICU interventions. These findings are similar to those from previous international studies of intensive care use by age [25, 41, 42], for cancer deaths compared with other causes [37, 41–43], and for ICU use overall [32]. We found that 6 % of deaths occurred during a hospital admission that included time in an ICU, which is similar to that reported in the UK and Belgium [25, 44], but lower than the approximately 20 % reported by large population-based studies in the USA [21, 24, 41, 44].

Fifty-five percent of deaths were recorded as occurring in a hospital or inpatient hospice. This is similar to results reported by other Australian studies, which also found that just over half of all deaths occurred in hospital [5–10] and that the proportions of deaths in hospital were higher for people dying from cancer [10] and lower for the very elderly [6, 7]. Internationally there is wide variation in the distribution of place of death, with the proportion of people dying in acute hospitals generally ranging from 45 to 67 % [13–15], and with the same trend of a lower proportion of the elderly dying in hospital [13, 15]. The importance of having detailed local information is exemplified by a study of people dying from cancer in London and New York, which found place of death varied significantly by patient and area characteristics [45]. Current information is also valuable, as, for example, it has been reported that the proportion of deaths occurring at home in the UK has varied from 31 % in 1974 to 18 % in 2003 and 21 % in 2010 [46, 47], although little variation was reported over the same period of time in a study of cancer deaths in NSW [3].

### Limitations

This study has some limitations. During the study period, the EDDC did not capture presentations to all EDs in NSW, so we were unable to provide a “whole-of-state” description of ED presentations during the last year of life. We therefore restricted the analyses to the area which we believed to have almost complete capture of ED presentations covering over 60 % of the NSW population. We could not reliably identify all people who were admitted to an inpatient hospice, as there were only five standalone inpatient hospices or inpatient palliative care units in NSW with their own institution codes. Admissions to inpatient hospice beds within larger hospital facilities could not be identified even though such beds are known to be available in a number of hospital facilities throughout NSW. Also, due to data not being made available, there was no coded cause of death information from the ABS for a substantial proportion of people who died in December 2007. However, we are relatively confident that this data limitation did not significantly affect our results, as the distribution of causes of death for those dying in December, where the data were available, was very similar to that for all other months, and the CCR data made it possible to identify a large number of cancer deaths for those with no information from the ABS Mortality data. Furthermore, when we excluded all people who died in December the results were not materially different to those reported. An additional limitation of the study is that restrictions on ABS Mortality data availability meant that we were limited to using data that are now around 8 years old. However, we suggest it would be unlikely that that there have been substantial changes in practice between 2006 - 2007 and the present day. Pleasingly, at the time of submission of this paper, more recent data were beginning to become available.

More generally, we cannot use these data sources to identify the “appropriateness” of care, as these data sources do not contain any patient-reported outcomes, nor do they address priorities and needs of the decedents or their carer(s). In addition, comorbid conditions are not comprehensively recorded in these data sets and so it is not possible to assess the potentially important impact of these conditions [48]. Nevertheless, the data from this study provide insights into patterns of hospital service utilisation for those dying in NSW hospitals, although little insight into the community care and services provided during the last year of life for either those who ultimately die in hospital or those who die in the community. Clearly access to community care dramatically impacts the experience of the last year of life and these data would be useful to complement our hospital data to provide a more comprehensive overview of dying in NSW. The multiple and variable community data sources for community care information are currently not readily accessible for record linkage studies. In addition to the identification of limitations in hospital-based information about palliative care specialist input, the data set did not allow for analysis of palliative care provided in the community setting, so we could not accurately describe primary and specialist palliative care service use in either of these settings. We contend that it is important to address these data gaps in order to provide more comprehensive descriptions of service use at the end of life, particularly palliative care, through administrative health datasets. This may serve to illuminate the discrepancies identified and any anomalous findings.

Finally, our analyses used data obtained through probabilistic record linkage, so it is possible that there were a small number of incorrect linkages of APDC or EDDC records, although the CHeReL estimated that there were only around 0.4 % false positive linkages and less than 0.5 % false negative linkages. We identified a small number of hospital admissions and ED presentations which could not be reconciled with the date of death, which if considered to be false positive linkages would equate to <0.1 % false positive linkages between the death data and hospital or ED records.

### Strengths

Despite these potential limitations, the data we have used provide powerful population-based information about end-of-life experiences and hospital services utilisation for an entire state during a full calendar year. This study includes all adult decedents, whereas most previous studies have been restricted to decedents aged over 65 years or with certain diseases. Our only exclusion was those aged less than 18 years, who comprise only 1 % of all deaths. This is the first study in Australia to describe use of hospital-based services at the end of life including numbers of hospitalisations and ICU use for all causes of death among all adults for a whole state, and only the second to report on place of death for all adult decedents (the first in NSW). While our results were reasonably similar to those from previous Australian studies of decedents aged over 65 years, the major difference was that, as we included all causes of death, we found different patterns of hospital and ED use [8], palliative care [10] and place of death [3] compared with studies examining specific causes such as cancer. For example, we found that people who die from cancer are more likely to spend more time in hospital and die in hospital than those dying from other causes. This more comprehensive description is important for understanding the different patterns of hospital-based end-of-life care and resource use. We have also included what we believe is the most comprehensive report to date of ED presentations, hospital-based palliative care and a description of hospital-based procedures undertaken at the end of life across NSW, giving an information-rich census of the use of these services towards the end-of-life. The data we used are being collected routinely and the linkage process is established and ongoing, which makes it possible to routinely update the summaries reported here and monitor activity over time, provided the coded cause of death information is available. These data will enable ongoing monitoring of the association between decedent characteristics (e.g. demographics, cause of death, residential and geographical indicators of socioeconomic disadvantage) and acute hospital-based services utilisation.

These data also provide a necessary foundation for asking the next set of questions. Such questions, for addressing in subsequent studies, would require a methodological approach other than record linkage. These could include investigating the reasons behind any differences identified in acute hospital-based services utilisation, whether and why some population groups encounter more barriers than others in service access and how all of this relates to the needs of individuals. With the foundation of this study, and the data from further studies addressing such questions, the planning of health services can be based around the goal of meeting clearly quantified needs and addressing identified barriers to service access.

## Conclusions

We found substantial differences in the utilisation of hospital-based services in the last year of life according to age, cause of death and place of residence. The results provide a necessary foundation for the planning of future health service needs and also help to identify areas for attention in the delivery and monitoring of end-of-life care. These existing health data collections give a highly informative description of hospital-based end-of-life experiences, providing a reliable, relatively inexpensive and ongoing source of population-wide information as well as insights into areas where the refining of data collection would assist in future analyses.

## Abbreviations

ABS: Australian Bureau of Statistics
APDC: Admitted Patient Data Collection
CCR: Central Cancer Registry
ED: Emergency Department
EDDC: Emergency Department Data Collection
ICU: Intensive care unit
IQR: Interquartile range
NSW: New South Wales
RBDM: Register of Births, Deaths and Marriages
